# Methodological Determinants of Grayscale Pixel Intensity in Ram Testicular Ultrasonography: Effects of ROI Placement and Imaging Plane Across Age Groups and Two Reproductive Periods

**DOI:** 10.1111/rda.70302

**Published:** 2026-08-04

**Authors:** Bülent Bülbül, Şükrü Doğan, Mustafa Yiğit Nizam, Necdet Akay

**Affiliations:** ^1^ Department of Reproduction and Artificial Insemination, Faculty of Veterinary Medicine Dokuz Eylül University İzmir Turkey; ^2^ Department of Animal Breeding Bahri Dağdaş International Agricultural Research Institute Karatay Turkey

**Keywords:** age, imaging plane, numerical pixel values, ram, reproductive periods, sampling region, testicular ultrasonography

## Abstract

Testicular ultrasonography is increasingly used in ram breeding soundness evaluations, yet methodological standardization of quantitative grayscale image analysis remains limited. The primary objective of this study was to determine the effects of region‐of‐interest (ROI) placement and imaging plane on testicular parenchymal numerical pixel values (NPVs) under a fixed ultrasonographic acquisition protocol, in Güney Karaman rams. A secondary objective was to compare NPV among age groups and between two predefined reproductive‐period sampling points, August and March. Twenty‐nine healthy, fertility‐proven rams were assigned to three age groups: 1.5 (*n* = 8), 3.1 (*n* = 12), and 4.6 years (*n* = 9). B‐mode ultrasonography was performed with a 7.5‐MHz linear transducer during the breeding (August) and non‐breeding season (March). From each testis, 10 standardized regions of interest were analysed (six longitudinal and four transversal, with five above and five below the mediastinum testis). ImageJ analysis yielded 1160 spot measurements. A linear mixed model with random intercepts for animal and testis ID accounted for the hierarchical repeated‐measures structure. NPVs increased with age, were higher in August than in March (71.65 ± 1.75 vs. 60.89 ± 1.75, *p* < 0.001), and were markedly higher below than above the mediastinum testis (89.50 ± 1.75 vs. 43.03 ± 1.75, *p* < 0.001). Transversal images yielded higher NPVs than longitudinal images (68.61 ± 1.77 vs. 63.93 ± 1.74, *p* < 0.001). Random‐effects analysis showed that 31%, 7%, and 62% of total variance were attributable to between‐animal, between‐testis, and spot‐level variation, respectively. These findings demonstrate that ram testicular echogenicity is influenced by biological and methodological factors, highlighting the need for standardized multi‐site sampling protocols.

## 1 Introduction

Reproductive performance is one of the most important parameters affecting flock profitability (Pardos et al. 2008). The reproductive capacity of rams plays a critical role in this performance (Mozo et al. 2015). MacLaren (1988) reported that approximately 50% of the reproductive potential of a flock is provided by the ram. Testicular examinations, referred to as the Breeding Soundness Examination (BSE), are performed to evaluate the ability of rams to mate with and impregnate ewes (Mozo et al. 2015). BSE may include anatomical and structural examinations, assessment of general health status, body condition scoring (BCS), testicular measurements, semen quality evaluation, and libido assessment (Kimberling and Parsons 2006).

In rams, reproductive performance is significantly affected by seasonal changes and they generally exhibit higher reproductive capacity during the breeding season (Aller et al. 2012; Sarlós et al. 2013; Palacios et al. 2016; Hedia et al. 2019). Seasonality is regulated by photoperiod and melatonin secretion (Malpaux et al. 1996), and may act as a limiting factor for reproduction in sheep (Chemineau et al. 2008).

Ultrasonography is a non‐invasive, non‐ionizing method that does not cause tissue damage. It is an indispensable clinical tool that provides real‐time and sequential information on male reproductive performance (Ortega‐Ferrusola et al. 2014; Gouletsou 2017). B‐mode ultrasonography has been used as a reliable method in various domestic animal species for estimating testicular volume (Brito et al. 2004; Zelli et al. 2013; Pozor et al. 2014), evaluating testicular parenchymal echotexture (Brito et al. 2003; Giffin et al. 2009; Abecia et al. 2020), and detecting early stages of macroscopic pathological processes or monitoring changes in lesions (Gouletsou 2017). Currently, ram testicular ultrasonography is performed using a computer‐assisted software. This system allows visual analysis of previously recorded B‐mode ultrasonographic images, referred to as ‘testicular ultrasonograms’ (Ahmadi et al. 2012; Camela et al. 2019; Brito et al. 2012). Ultrasonograms consist of small image units called pixels, and variations in grey‐scale pixel intensity provide information about tissue density (Giffin et al. 2009; Camela et al. 2019). In computer‐assisted image analysis, the numerical values of testicular echogenicity, known as ‘pixel intensity’, range from 0 to 255, where 0 represents absolute black and 255 represents absolute white. Testicular heterogeneity is determined by calculating the standard deviation of pixel values (Arteaga et al. 2005; Giffin et al. 2009; Brito et al. 2012; Kumari et al. 2015; Moxon et al. 2015). The primary aim of testicular ultrasonography is not only to identify males with testicular pathology (Ahmad et al. 2000), but also to predict seminal quality and, consequently, the fertility of breeding males. In this context, while some previous studies conducted on rams have reported moderate correlations between testicular blood flow, echotextural characteristics, and sperm quality (Ntemka et al. 2018; Hedia et al. 2019, 2020), other studies have reported inconsistent or non‐significant associations between testicular echotexture and semen quality or breeding soundness indicators, including adult Texel rams (Urt et al. 2018) and Nelore bulls (Pinho et al. 2013).

Despite the increasing use of computer‐assisted ultrasonographic image analysis to quantify testicular echotexture in rams, methodological aspects of image acquisition and region‐of‐interest (ROI) selection remain incompletely standardized. This is relevant because grey‐scale ultrasonographic echotexture is influenced by acoustic tissue interfaces, vascularity, and the structural organization of the testicular parenchyma (Giffin et al. 2009). In rams, quantitative ultrasonographic image attributes have also been associated with testicular tissue composition, supporting the biological relevance of pixel‐based echotextural analysis (Ahmadi et al. 2013). Moreover, correlations between ultrasonographic and microscopic testicular characteristics in ram lambs indicate that echotextural parameters may reflect underlying spermatogenic and parenchymal organization (Giffin et al. 2014). However, previous studies have used different analytical strategies, including selected parenchymal areas, minimum representative‐area approaches, seasonal pixel‐intensity assessment, and ultrasound‐video analysis platforms (Urt et al. 2018; Hedia et al. 2020; Carvajal‐Serna et al. 2022). Similar ROI‐based computer‐assisted approaches have also been applied in other farm animal species, in which pixel intensity and echotexture heterogeneity were calculated from selected testicular parenchymal areas (El‐Sherbiny et al. 2023). In addition, the mediastinum testis is a distinct ultrasonographic structure whose echogenicity and thickness may vary with age, suggesting that the anatomical position of sampling areas within the testicular parenchyma deserves methodological consideration (Andrade et al. 2014). Therefore, systematic evaluation of the intratesticular sampling region and imaging plane may improve the reproducibility and comparability of testicular echotexture measurements in rams.

Although ultrasonography is considered a potential tool for evaluating testicular parenchymal echotexture and providing important information on testicular function and reproductive potential in rams, studies examining the relationship between ultrasonogram pixel intensity values and age or reproductive period variations are still limited. Therefore, the primary objective of this study was to determine the effects of ROI placement and imaging plane on testicular parenchymal NPV under a fixed ultrasonographic acquisition protocol. A secondary objective was to compare NPV among age groups and between two predefined reproductive‐period sampling points, August and March.

## 2 Material and Methods

### 2.1 Animals

The study was conducted at the Bahri Dağdaş International Agricultural Research Institute, Konya, Turkey, located in the northern hemisphere (1016 m above sea level, 37.857063 north latitude and 32.567036 east longitude). The study location is considered to be in the breeding season in August and the non‐breeding season in March (Arikan et al. 2021; Kırbaş et al. 2025; Üstüner et al. 2014). In the study, 29 mature, fertility‐proven, single‐born Güney Karaman rams of different ages, with a live weight of 45–55 kg, were used. The rams were allocated to 3 groups according to their ages at examination in August (with an ambient temperature of 24.0°C (17.2°C–30.9°C) and sunny hours of 10.8 h/day). The rams were 1.50 ± 0.027 (range 1.4–1.6 years) years old in group 1 (*n* = 8), 3.10 ± 0.137 (range 2.5–3.5 years) years old in group 2 (*n* = 12), and 4.63 ± 0.109 (range 4.5–5.5 years) years old in group 3 (*n* = 9) (mean ± SEM, *p* < 0.001) at the beginning of the study (August). The mean birth weights were 3.837 ± 0.171, 3.569 ± 0.201, and 3.833 ± 0.198 kg in age groups 1, 2, and 3, respectively (mean ± SEM, *p* = 0.529). The same rams were examined again 6 months later, in March (with an ambient temperature of 6.0°C (0.2°C–12.5°C) and sunny hours of 6.3 h/day).

The animals were housed in barns under standardized conditions for housing, lighting, and feeding throughout the study. They were fed according to the nutritional requirements stated by the National Research Council (2007). Additional supplements and water were also given ad libitum. All rams remained healthy throughout the study period. Before image analysis, the testes were assessed for clinically and ultrasonographically detectable abnormalities. Images showing discrete focal hyperechoic lesions with acoustic shadowing, cystic areas, marked parenchymal distortion, or other overt abnormalities were not considered suitable for quantitative parenchymal analysis.

### 2.2 Testicular Ultrasonography

**FIGURE 1:**
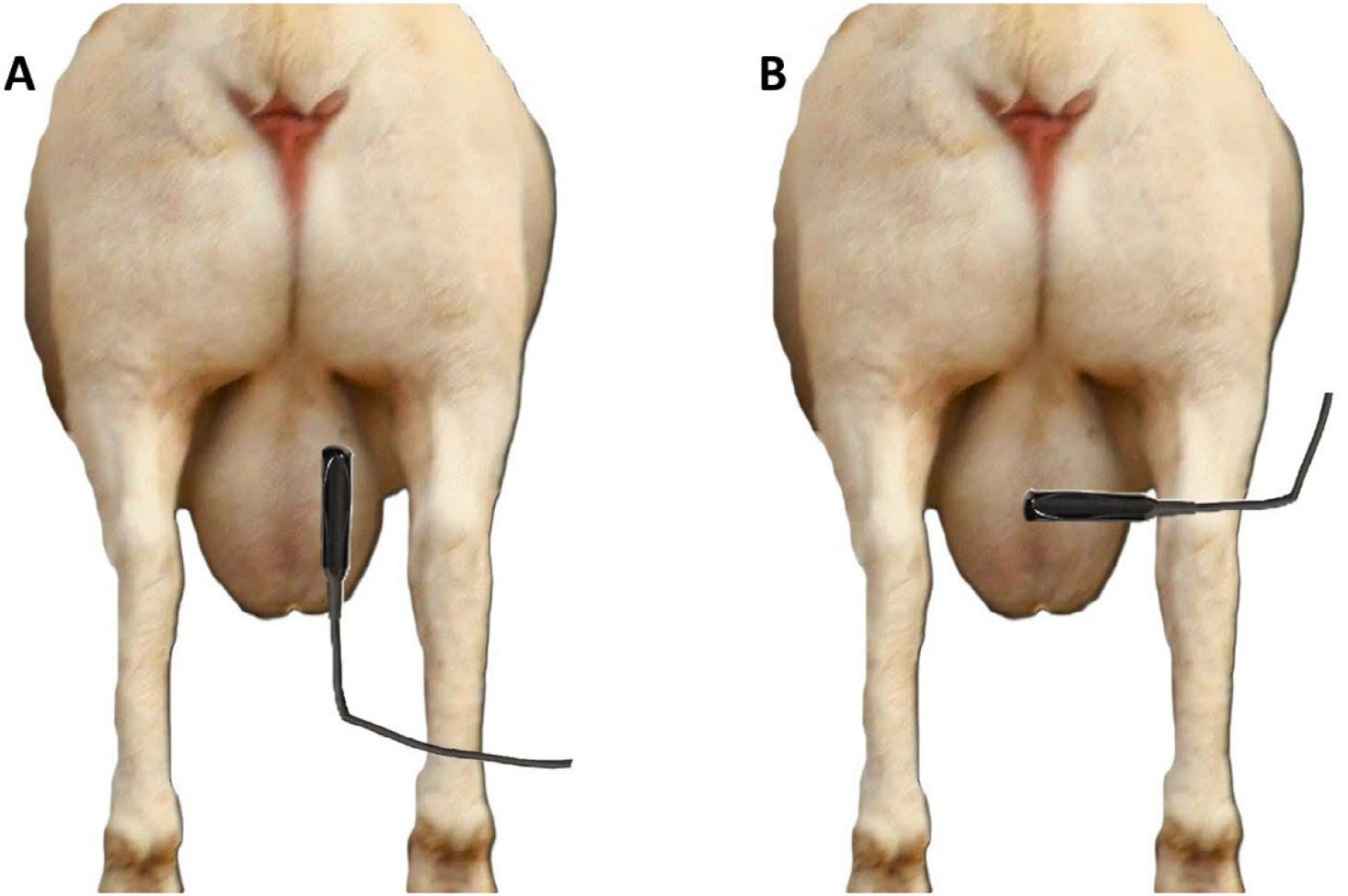
Placement of the probe for the longitudinal (A) and transversal (B) USG examinations.

**FIGURE 2:**
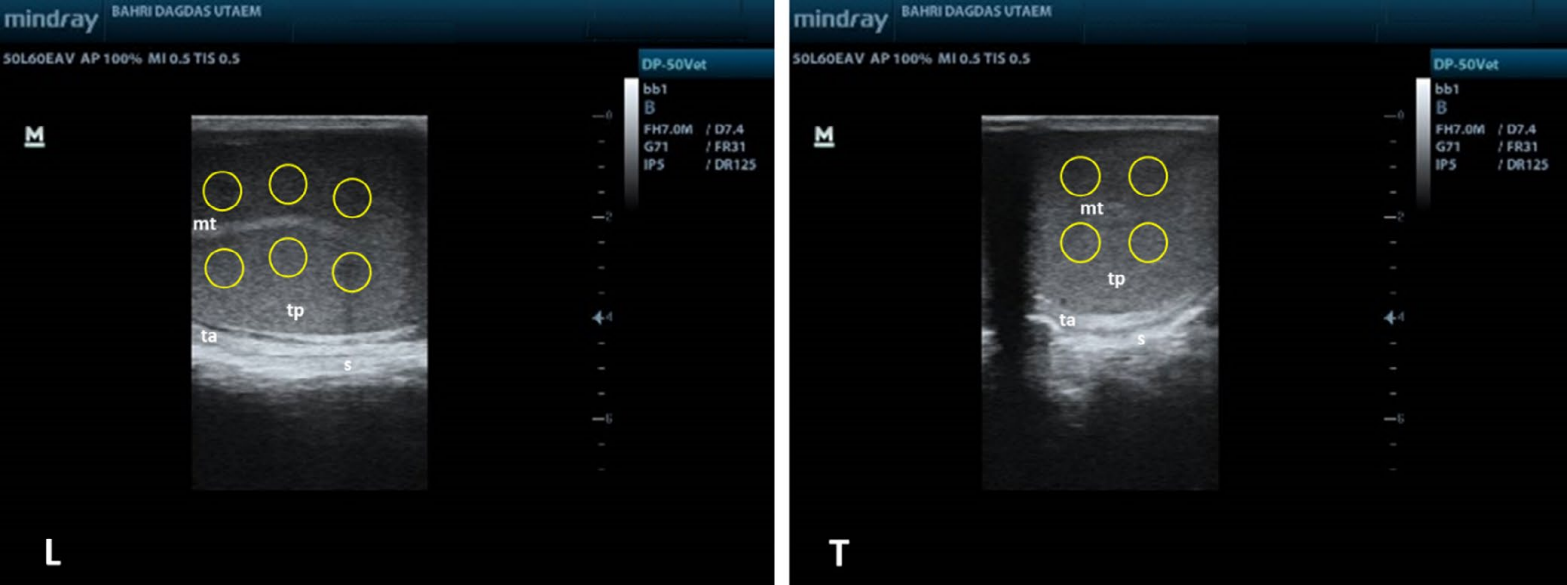
The longitudinal and transversal USG imaging planes used for computer‐assisted echotextural analysis. The circles (six for longitudinal and four for transversal planes; 3 spots above and 3 spots below, and 2 spots above and 2 spots below the mediastinum testis, respectively for longitudinal and transversal planes) illustrate the sampling regions for the computer‐generated spot meters that are used to assess numerical pixel values (NPVs) and are not intended to indicate exact geometrical distances from the mediastinum testis. L, longitudinal; mt, mediastinum testis; s, scrotum; T, transversal; ta, *Tunica albuginea*; tp, testicular parenchyma.

Testicular ultrasonography was performed on the same day using a B‐mode ultrasound scanner with a 7.5‐MHz linear transducer (Mindray DP‐50 VET, Shenzhen Mindray Bio‐Medical Electronics Co Ltd., Shenzhen, China) in all groups. All evaluations were performed by a single professional, and all settings on the ultrasound unit, including the range, contrast, overall gain, near and far gain, and focal depth (4 cm), were kept constant throughout the examinations. Rams were physically restrained by two assistants while the testes were immobilized without pressure. As previously described (Andrade et al. 2014), the USG examinations were performed by standing behind the ram and approaching the testes from the caudal end. In brief, the long hairs on the scrotum were first trimmed with scissors. Then, an aqueous gel was used as a coupling material between the transducer and the testes. Scans were performed in the longitudinal and transversal planes to evaluate the testicular parenchyma (Figure 1), as previously described by Ahmadi et al. (2013). Briefly, images of the largest cross‐sectional area of each testis were captured as still images and recorded on the ultrasound device's hard disk. To ensure the integrity of our data, all recorded images were carefully screened before proceeding with image analysis. Only those that were completely free of artefacts and captured the entire testis in full detail were selected for further evaluation. Computerized image analysis of the testicular parenchyma was conducted using an image analytical software (ImageJ 1.48v, Wayne Rasband National Institutes of Health, USA). Using the spot meter technique, 10 spots of ~1 cm^2^ each for each testis (six for longitudinal and four for transversal planes; three spots above and three spots below, and two spots above and two spots below the mediastinum testis, respectively, for longitudinal and transversal planes) were placed. To ensure consistency, the spots (six for longitudinal and four for transversal planes) were standardized and fixed, and then carefully overlaid onto the images, keeping the mediastinum perfectly centred within the spots. The ROIs were standardized according to their number, approximate area, imaging plane, and location above or below the mediastinum testis. ROIs were placed within artefact‐free parenchyma, without overlap or inclusion of the mediastinum. Exact mirror symmetry based on measured pixel distances from the mediastinum was not imposed because the available parenchymal area and testicular contour varied among images. Therefore, the terms ‘above’ and ‘below’ describe the sampling regions relative to the mediastinum in the stored ultrasonographic image rather than geometrically equidistant regions. The mean NPV from each ROI was recorded. The grey values obtained from 20 spots (10 from each testis) for one ram were recorded for statistical analysis (Figure 2).

### 2.3 Statistical Analyses

All statistical analyses were performed using IBM SPSS Statistics for Windows (version 23.0; IBM Corp., Armonk, NY, USA). The significance level was set at *α* = 0.05 for all tests. Normality of residuals was assessed using Shapiro–Wilk tests and visual inspection of *Q*–*Q* plots of the standardized residuals. Homogeneity of variances was assessed using Levene's test.

#### 2.3.1 Data Structure

The experimental design involved repeated measurements on the same animals across two reproductive periods (breeding and non‐breeding, in August and March), with multiple spot measurements per testis. Specifically, each ram contributed 20 spot measurements per reproductive period (10 spots from the right testis and 10 from the left testis), resulting in a hierarchical data structure: spots nested within testes, testes nested within animals, and repeated measures across seasons. To account for these dependencies and the repeated measures, a linear mixed model (LMM) approach was employed.

#### 2.3.2 Model Building Strategy

A stepwise backward elimination approach was adopted for model selection, based on the statistical significance of fixed effects (*p* > 0.05 as the removal criterion). Initially, a full model was fitted, including all main effects (AgeGroup, Season, ROI, and Plane) and all possible two‐way and three‐way interaction terms.

The full model revealed that the following interaction terms were not statistically significant and were therefore removed sequentially:

- Season × Plane (*p* = 0.991).
- ROI × Plane (*p* = 0.492).

Although AgeGroup × Plane was statistically significant (*p* = 0.018), its effect size was small, and its inclusion did not materially alter the estimates or significance of other fixed effects in the model. Its inclusion did not result in meaningful changes to the estimates of primary biological interest, and it was excluded to ensure model stability and parsimony. Moreover, the study focused primarily on the effects of age, season, and ROI. While the AgeGroup × Plane interaction was excluded in the interest of parsimony, Plane was retained as a fixed effect to account for its potential influence on the main estimates.

The three‐way interaction AgeGroup × Season × ROI was significant (*p* = 0.018) and retained in the final model, as it provided meaningful biological insight into the combined effects of age, season, and ROI on testicular echogenicity.

Thus, the final parsimonious model included all main effects (AgeGroup, Season, ROI and Plane) and the following interaction terms: AgeGroup × Season, AgeGroup × ROI, Season × ROI, and AgeGroup × Season × ROI.

#### 2.3.3 Final Linear Mixed Model

The final parsimonious model included the following fixed effects:

- AgeGroup (three levels: Group 1, Group 2 and Group 3).
- Season (two levels: breeding, non‐breeding).
- ROI (two levels: above mediastinum, below mediastinum).
- Plane (two levels: longitudinal, transversal).
- Two‐way interactions: AgeGroup × Season, AgeGroup × ROI, Season × ROI.
- Three‐way interaction: AgeGroup × Season × ROI.

Random effects were specified to account for the hierarchical data structure:

- A random intercept for Animal ID (accounting for between‐animal variation).
- A random intercept for Testis ID nested within Animal ID (accounting for between‐testis variation within the same animal and for the clustering of the 10 spot measurements within each testis).

#### 2.3.4 Covariance Structure

The covariance structure for random effects was specified as variance components (VC). Although autoregressive (AR1) structures are sometimes used for repeated measures, our study involved only two time points (August and March), in which case AR1 reduces to compound symmetry and offers no advantage over VC. Furthermore, spot measurements within each testis were obtained simultaneously without temporal ordering, making AR1 inappropriate at that level. Therefore, VC was selected as the most parsimonious and appropriate structure.

The model was fitted using restricted maximum likelihood (REML) estimation, which provides unbiased estimates of variance components.

The general form of the final model was:

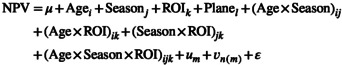

where *u_m_* ~ *N*(0, *σ* ^2^_Animal) and *v_n_* ₍*m*₎ ~ *N*(0, *σ* ^2^_Testis).

#### 2.3.5 Post Hoc Comparisons

For significant fixed effects and interactions, post hoc pairwise comparisons were performed using estimated marginal means (EMMEANS) with the Bonferroni correction for multiple comparisons. The results are presented in Table 3 using superscript letters. Within each panel, means with different superscript letters differ significantly (Bonferroni‐adjusted *p* < 0.05).

#### 2.3.6 Effect Sizes and Confidence Intervals

Group comparisons are based on EMMEANS presented in Table 3, with 95% confidence intervals reported alongside the means.

#### 2.3.7 Model Diagnostics

Model assumptions were evaluated as follows:

- Normality of residuals was assessed using Shapiro–Wilk tests and visual inspection of histograms and *Q*–*Q* plots of the standardized residuals.
- Homoscedasticity (constant variance of residuals) was evaluated by plotting standardized residuals against predicted values.
- The presence of influential observations was examined using Cook's distance values (values > 1 were considered potentially influential).

#### 2.3.8 Reporting

Results are reported as estimated marginal means ± standard error of the mean (SEM) unless otherwise specified. All statistical tests were two‐tailed. A *p*‐value of less than 0.05 was considered statistically significant; actual *p*‐values are reported where possible, with values less than 0.001 reported as *p* < 0.001.

## 3 Results

### 3.1 Descriptive Statistics

A total of 1160 spot measurements were analysed from 29 Güney Karaman rams across two seasons (reproductive periods). Descriptive statistics of NPVs stratified by age group and season are presented in Table 1. During the breeding season, mean NPV increased with age, with Group 3 showing the highest value (83.05 ± 2.41), followed by Group 2 (69.98 ± 2.06) and Group 1 (60.50 ± 2.03). During the non‐breeding season, Group 3 again had the highest mean NPV (65.69 ± 1.91), while Group 2 (55.91 ± 1.81) showed lower values than Group 1 (59.67 ± 1.71). Overall, the mean NPV was higher during the breeding season (71.42 ± 1.31) than during the non‐breeding season (59.98 ± 1.08).

**TABLE 1:** Descriptive statistics (mean ± SEM) of NPV in Güney Karaman rams by AgeGroup and season (breeding and non‐breeding).

AgeGroup	*n* (rams)	*n* (spots)	Mean NPV	SD	SEM
Group 1					
Breeding	8	160	60.50	25.62	2.03
Non‐breeding	8	160	59.67	21.57	1.71
Group 2					
Breeding	12	240	69.98	31.93	2.06
Non‐breeding	12	240	55.91	27.99	1.81
Group 3					
Breeding	9	180	83.05	32.30	2.41
Non‐breeding	9	180	65.69	25.60	1.91
Overall					
Breeding	29	580	71.42	31.62	1.31
Non‐breeding	29	580	59.98	25.90	1.08

Abbreviation: NPV, numerical pixel value.

### 3.2 Linear Mixed Model Results

The final linear mixed model, selected after backward elimination of non‐significant interactions, included all main effects (AgeGroup, Season, ROI, Plane) and the two‐way interactions AgeGroup × Season, AgeGroup × ROI, Season × ROI, as well as the three‐way interaction AgeGroup × Season × ROI. Random intercepts were specified for Animal ID and Testis ID (nested within Animal ID). The covariance structure was a VC model, and the model was fitted using REML.

The Type III tests of fixed effects (Table 2) revealed significant main effects for all factors: AgeGroup (*F*_2,26_ = 6.17, *p* = 0.006), Season (*F*_1,1092_ = 225.17, *p* < 0.001), Location (*F*_1,1092_ = 4203.49, *p* < 0.001), and Plane (*F*_1,1092_ = 42.13, *p* < 0.001). All two‐way interactions were highly significant (*p* < 0.001 for each), and the three‐way interaction (AgeGroup × Season × ROI) was also significant (*F*_2,1092_ = 4.02, *p* = 0.018).

**TABLE 2:** Type III tests of fixed effects for the final linear mixed model.

Source	Numerator df	Denominator df	*F*	*p*
AgeGroup	2	26	6.17	**0.006**
Season	1	1092	225.17	**< 0.001**
ROI	1	1092	4203.49	**< 0.001**
Plane	1	1092	42.13	**< 0.001**
AgeGroup × Season	2	1092	44.93	**< 0.001**
AgeGroup × ROI	2	1092	14.86	**< 0.001**
Season × ROI	1	1092	61.34	**< 0.001**
AgeGroup × Season × ROI	2	1092	4.02	**0.018**

*Note:* Dependent variable: NPV. Denominator degrees of freedom calculated using the Satterthwaite approximation. Values presented in bold were considered statistically significant (*p* < 0.05).

Abbreviations: NPV, numerical pixel value; ROI, region of interest.

### 3.3 Estimated Marginal Means

**FIGURE 3:**
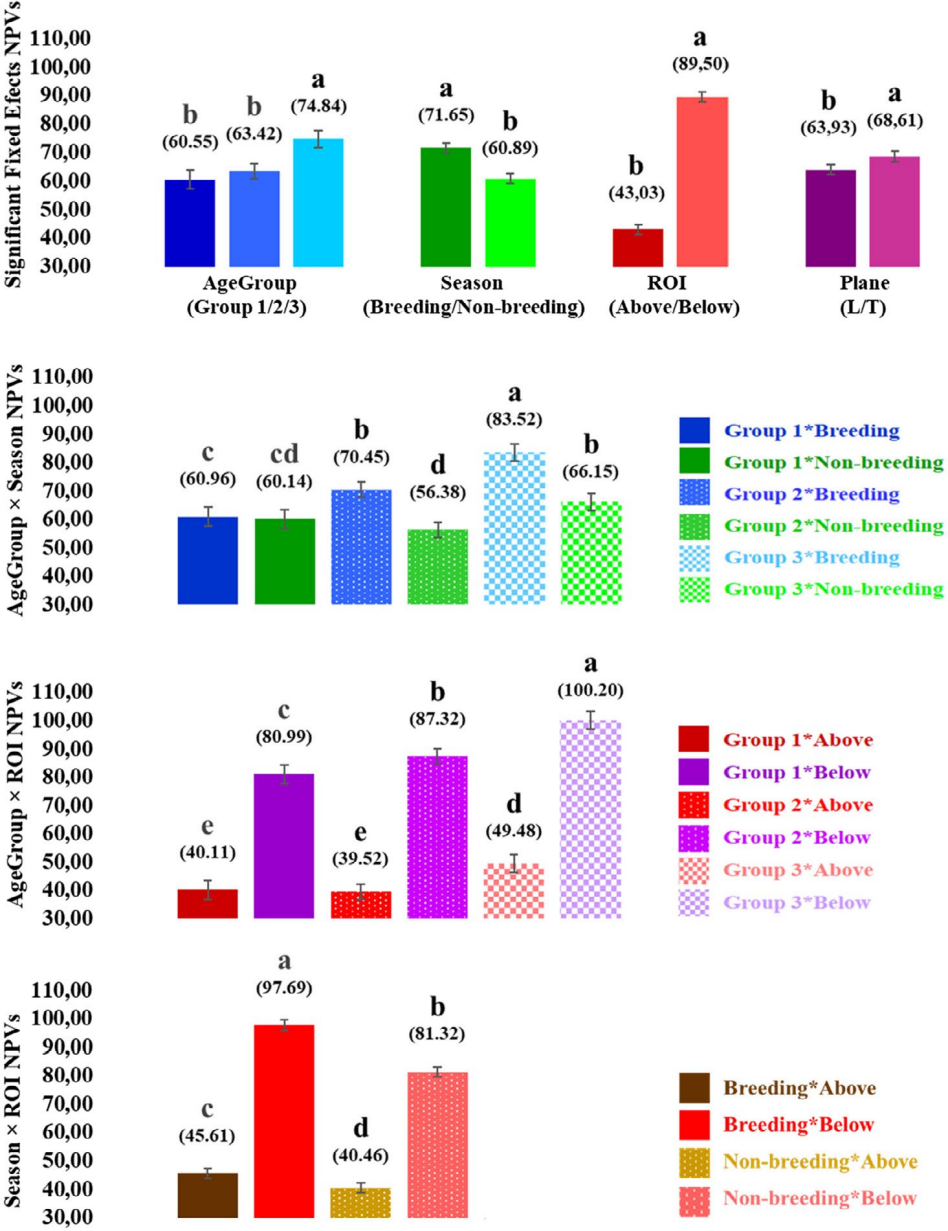
The EMMEANS for significant effects and interactions (AgeGroup × Season, AgeGroup × ROI, and Season × ROI). ^a–e^Means with different superscripts within each parameter differ significantly (*p* < 0.05). EMMEANS, estimated marginal means; L, longitudinal; NPV, numerical pixel value; ROI, region of interest; T, transversal.

The EMMEANS for all significant effects and interactions are presented in Tables 3 and S1, and Figure 3. Within each panel of the table, means with different superscript letters differ significantly (Bonferroni‐adjusted *p* < 0.05).

**TABLE 3:** The EMMEANS of NPVs for significant fixed effects and interactions.

Factor/interaction	Mean NPV	SE	95% CI
AgeGroup			
Group 1	60.55^b^	3.22	53.94–67.16
Group 2	63.42^b^	2.63	58.02–68.81
Group 3	74.84^a^	3.03	68.61–81.07
Season			
Breeding	71.65^a^	1.75	68.06–75.23
Non‐breeding	60.89^b^	1.75	57.31–64.48
ROI			
Above mediastinum	43.03^b^	1.75	39.45–46.62
Below mediastinum	89.50^a^	1.75	85.92–93.09
Plane			
Longitudinal	63.93^b^	1.74	60.37–67.49
Transversal	68.61^a^	1.77	64.99–72.22
AgeGroup × Season			
Group 1 × Breeding	60.96^d^	3.29	54.24–67.69
Group 1 × Non‐breeding	60.14^d^	3.29	53.41–66.86
Group 2 × Breeding	70.45^b^	2.68	64.96–75.94
Group 2 × Non‐breeding	56.38^e^	2.68	50.89–61.87
Group 3 × Breeding	83.52^a^	3.10	77.18–89.86
Group 3 × Non‐breeding	66.15^c^	3.10	59.81–72.49
AgeGroup × ROI			
Group 1 × Above	40.11^e^	3.29	33.38–46.83
Group 1 × Below	80.99^c^	3.29	74.27–87.72
Group 2 × Above	39.52^e^	2.68	34.02–45.01
Group 2 × Below	87.32^b^	2.68	81.82–92.81
Group 3 × Above	49.48^d^	3.10	43.14–55.82
Group 3 × Below	100.20^a^	3.10	93.86–106.54
Season × ROI			
Breeding × Above	45.61^c^	1.82	41.90–49.31
Breeding × Below	97.69^a^	1.82	93.98–101.39
Non‐breeding × Above	40.46^d^	1.82	36.76–44.17
Non‐breeding × Below	81.32^b^	1.82	77.61–85.03

*Note:* Within each panel, means with different superscript letters differ significantly (Bonferroni‐adjusted *p* < 0.05).

Abbreviations: EMMEANS, estimated marginal means; NPV, numerical pixel value; ROI, region of interest.

**FIGURE 4:**
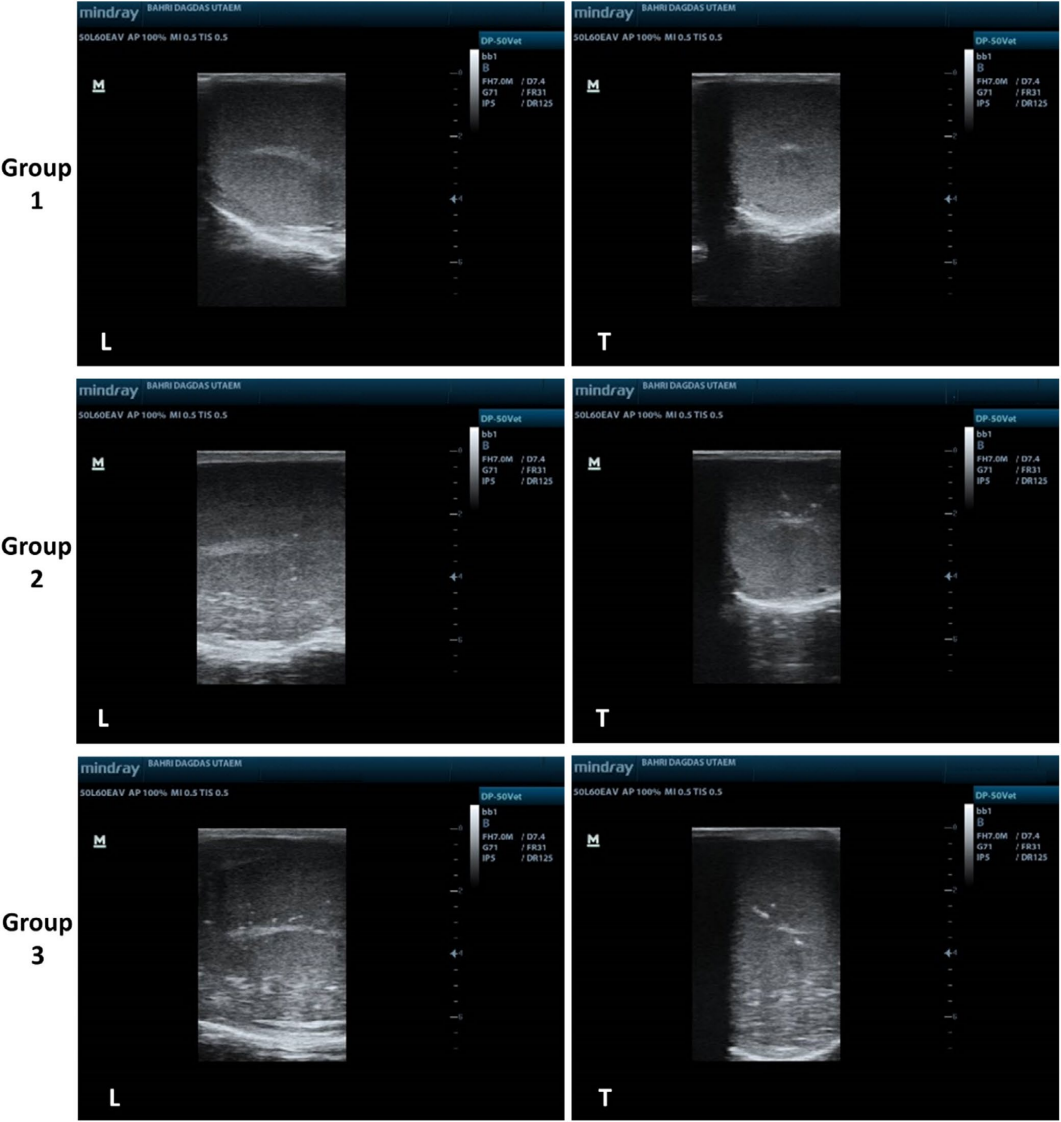
The longitudinal and transversal USG examination examples obtained in AgeGroups in the breeding season. L, longitudinal; T, transversal.

The NPV increased with age, with Group 3 having the highest mean (74.84 ± 3.03) and Group 1 the lowest (60.55 ± 3.22) (Figure 4). Breeding season NPV (71.65 ± 1.75) was higher than non‐breeding NPV (60.89 ± 1.75). The below region of mediastinum (89.50 ± 1.75) showed markedly higher NPV than the region above (43.03 ± 1.75). The transversal plane (68.61 ± 1.77) yielded a higher NPV than the longitudinal plane (63.93 ± 1.74).

The Season × ROI interaction revealed that the difference between below and above the mediastinum was greater during the breeding season (97.69 vs. 45.61) compared to the non‐breeding season (81.32 vs. 40.46). The AgeGroup × ROI interaction showed that in the below‐mediastinum region, Group 3 had the highest NPV (100.20), followed by Group 2 (87.32) and Group 1 (80.99). Within the above mediastinum region, NPV values were similar across age groups (range: 39.52–49.48).

The three‐way interaction (AgeGroup × Season × ROI) further demonstrated that the highest NPV values were observed in Group 3 during the breeding season below the mediastinum (112.94 ± 3.23), while the lowest values were in Group 2 during the non‐breeding season above the mediastinum (35.41 ± 2.79).

### 3.4 Random Effects and Model Fit

The random effects analysis (Table 4) revealed significant variance components for both Animal ID (*σ* ^2^ = 71.34, *p* = 0.002) and Testis ID (*σ* ^2^ = 15.45, *p* = 0.010), confirming the appropriateness of the hierarchical model. The intraclass correlation coefficient (ICC) for Animal ID was 0.308, indicating that approximately 31% of the total variance in NPV was attributable to differences among individual rams. The ICC for Testis ID was 0.375, indicating that 38% of the total variance was explained by differences between animals and between testes within animals (i.e., the combination of animal‐level and testis‐level variation). The remaining 62% of the variance was due to residual error at the spot level, suggesting that substantial biological and/or technical variation exists even among different ROIs of the same testis.

**TABLE 4:** Estimates of covariance parameters for random effects.

Random effect	Variance (*σ* ^2^)	SE	Wald *Z*	*p*	% of total variance	ICC
Animal_ID	71.34	23.12	3.09	0.002	30.8%	0.308
Testis_ID (nested within animal)	15.45	5.96	2.59	0.010	6.7%	0.375*
Residual	144.72	6.19	23.37	< 0.001	62.5%	—
Total	231.51				100%	

*Note:* *ICC for Testis represents the proportion of variance explained by animal + testis combined (37.5%). ICC for Animal = *σ* ^2^_Animal/(*σ* ^2^_Animal + *σ* ^2^_Testis + *σ* ^2^_Residual) = 71.34/(71.34 + 15.45 + 144.72) = 0.308; ICC for Testis = (*σ* ^2^_Animal + *σ* ^2^_Testis)/(*σ* ^2^_Animal + *σ* ^2^_Testis + *σ* ^2^_Residual) = (71.34 + 15.45)/231.51 = 0.375; total variance = 231.51.

## 4 Discussion

The present study demonstrates that testicular parenchymal NPVs in Güney Karaman rams are influenced not only by biological factors such as age and reproductive period, but also by methodological factors related to image sampling. Although older rams and the reproductive period were associated with higher NPV values, the most prominent finding was the marked effect of region‐of‐interest (ROI) placement and imaging plane. In particular, the difference between regions above and below the mediastinum testis was approximately 46.5 NPV units, with mean values of 43.03 and 89.50, respectively. The effect of the imaging plane was smaller but still evident, with mean values of 63.93 in longitudinal images and 68.61 in transversal images. These findings indicate that testicular echotexture values cannot be interpreted independently of where and how the measurement is obtained.

The biological relevance of computer‐assisted testicular echotexture analysis has been supported by previous studies in rams. Giffin et al. (2009) showed that ram testicular NPVs were associated with seminiferous tubule microstructure and that echotextural values are affected by the interaction of ultrasound beams with acoustic tissue interfaces. Ahmadi et al. (2013) further reported quantitative relationships between testicular image attributes and the biochemical composition of ram testicular tissue. Similarly, Giffin et al. (2014) demonstrated associations between ultrasonographic echotexture and microscopic characteristics during testicular development in ram lambs. Therefore, NPV is not merely an arbitrary image value; it may reflect underlying testicular structure. However, the present findings show that this biological interpretation is only meaningful when methodological variation is controlled.

Age had a significant effect on NPV, with Group 3 showing the highest estimated mean value (74.84 ± 3.03), followed by Group 2 (63.42 ± 2.63) and Group 1 (60.55 ± 3.22). Higher NPV observed in older age groups is consistent with previous observations that testicular echogenicity changes with maturation. Andrade et al. (2014) reported that testicular parenchymal echogenicity increased with age in Santa Inês lambs and that the mediastinum testis also became more echogenic and thicker over time. Camela et al. (2019) showed that pubertal status was associated with changes in testicular echotexture and pixel heterogeneity in Dorper rams; however, peripubertal rams had higher NPVs than postpubertal rams in their study. This indicates that the direction of age‐related echotextural change may vary according to breed, age range, reproductive status, climate, and analytical protocol. Thus, the higher NPV observed in older Güney Karaman rams may reflect maturational changes in parenchymal organization and testicular histophysiology, but age‐related NPV patterns should not be generalized without methodological and biological context.

In our study, NPVs differed between the reproductive periods under the current environmental and management conditions. The estimated mean NPV was higher in August (71.65 ± 1.75) than that in March (60.89 ± 1.75), corresponding to a difference of approximately 10.8 NPV units. This confirms that testicular echogenicity is a dynamic variable and may change with seasonal reproductive physiology. Although their study was conducted in a subtropical location (Egypt, with ambient temperatures ranging from 20.5°C to 33°C, and sunny hours ranging from 11 to 14 h/day), which has different seasonal characteristics from our study location, our results are consistent with those of Hedia et al. (2020), who reported significant seasonal variation in testicular pixel intensity in Awassi rams. They also found significant associations between pixel intensity and semen characteristics, suggesting that ultrasonographic appearance may reflect seasonal modulation of testicular function. Ntemka et al. (2018) also showed that relationships among testicular echogenicity, hemodynamic indices, and semen parameters may depend on season and age. Therefore, season may be considered an important biological covariate in ram testicular echotexture studies, but the direction and magnitude of seasonal NPV changes may differ across breeds, latitudes, photoperiods, ambient temperatures, management, and definitions of breeding and non‐breeding periods.

The main methodological finding of this study was the ROI effect. The mean NPV measured below the mediastinum was more than twice that measured above the mediastinum, with estimated values of 89.50 ± 1.75 and 43.03 ± 1.75, respectively. This difference was much larger than the age, reproductive period, and plane effects. Therefore, ROI placement is not a minor technical choice; it is a major source of measurement variation. If one study samples predominantly above the mediastinum and another samples below it, their NPV results may differ substantially even if the biological status of the testes is similar. For this reason, ROI placement relative to the mediastinum testis must be standardized or explicitly reported in ram testicular echotexture studies. In this study, the mediastinum testis was used as an anatomical landmark rather than as an internal grayscale reference. Although parenchyma‐to‐mediastinum ratios may reduce equipment‐related variability, the mediastinum may itself change in echogenicity and thickness with age (Andrade et al. 2014). A ratio based on a biologically variable denominator could therefore obscure age‐ or period‐associated effects. Future studies, particularly in multicentre or multi‐device settings, should compare the repeatability and biological validity of absolute and mediastinum‐normalized NPV measurements.

This finding is particularly important because ROI strategies have not yet been fully standardized, and thus, previous studies have used different approaches. Ahmadi et al. (2013) used circular ROIs above and below the rete testis in longitudinal and transverse planes. Giffin et al. (2009) used spot meters distributed around the rete testis, and Giffin et al. (2014) used multiple spots in longitudinal and transverse views, with an equal number placed above and below the rete testis. In contrast, Hedia et al. (2020) used a single selected rectangular area in apparently homogeneous parenchyma, while Urt et al. (2018) evaluated different representative areas for echotexture assessment. These methodological differences may partly explain why published associations between testicular echotexture and semen or reproductive parameters are not always consistent. The present study directly shows that ROI placement alone can produce a difference of nearly 47 NPV units, which is large enough to alter biological interpretation.

The imaging plane also influenced NPV. Transversal images produced higher NPVs than longitudinal images, with estimated means of 68.61 ± 1.77 and 63.93 ± 1.74, respectively. Although this difference was smaller than the ROI effect, it was statistically significant and methodologically relevant. Since ultrasound echogenicity depends on reflection, scattering, beam angle, tissue depth, and acoustic interfaces, longitudinal and transversal planes may not capture the same parenchymal characteristics in the same way. Giffin et al. (2009) emphasized that quantitative echotextural attributes are affected by acoustic tissue interfaces and attenuation. In the present study, machine settings were kept constant, so the plane effect most likely reflects differences in anatomical orientation and beam–tissue interaction rather than device adjustment. Therefore, longitudinal and transversal NPV measurements should not be pooled or compared without considering the imaging plane as a methodological factor.

The relatively high residual variance (62.5%) reflects substantial variation at the spot level within the same testis. This is not unexpected, as testicular parenchyma is inherently heterogeneous; local echogenicity can vary depending on seminiferous tubule density, proximity to the mediastinum, and localized acoustic shadowing artefacts (Ahmad et al. 1993). The significant fixed effects of ROI and Plane in Table 2 confirm that part of this residual variation is systematically explained by these anatomical and technical factors. For instance, the imaging plane can influence which specific structural features or vascular networks are most prominently reflected in the echotexture. However, even after accounting for these factors, considerable spot‐level variation remains. This finding aligns with veterinary ultrasonography principles, indicating that a single measurement is insufficient to represent the entire parenchyma, and multiple sampling sites per testis are strictly necessary for reliable and reproducible echotexture assessment (Pozor et al. 2017).

The variance structure further supports the need for standardized multi‐ROI sampling. Random‐effects analysis showed that 30.8% of the total variance was attributable to animal‐level differences and 6.7% to testis‐level differences, while 62.5% remained at the spot level. This means that most of the variation occurred among individual measurement sites rather than only among animals or testes. Therefore, a single ROI cannot reliably represent the entire testicular parenchyma. A structured multi‐ROI protocol is necessary to reduce sampling error and improve reproducibility. This is consistent with previous ram studies that used multiple spot meters and with Urt et al. (2018), who specifically addressed the minimum representative area required for testicular echotexture assessment.

The functional interpretation of NPV should therefore be cautious. Several studies suggest that testicular echotexture may be related to semen quality or reproductive function. Carvajal‐Serna et al. (2022) reported associations between ultrasound‐video echotexture parameters and sperm quality traits in rams, while Montes‐Garrido et al. (2022) showed that semen collection frequency influenced testicular echotexture, Doppler parameters, and sperm quality. However, some other studies have reported inconsistent or negative associations. Urt et al. (2018) found no relationship between testicular echotexture and seminal characteristics in adult Texel rams, and Pinho et al. (2013) reported no relationship between testicular pixel intensity and breeding soundness indicators in adult Nelore bulls. These discrepancies reinforce the central message of the present study: before NPV can be used confidently in functional reproductive models, its measurement protocol must be standardized.

The present study has several important strengths. The same animals were evaluated across reproductive periods, all ultrasonographic settings were kept constant, examinations were performed by a single operator, and a systematic spot‐meter approach was used in both longitudinal and transversal planes. These features reduce technical variability and increase the likelihood that the observed differences reflect biological and sampling‐related variation rather than operator‐dependent or device‐dependent artefacts. In addition, the use of a mixed‐model approach allowed the hierarchical structure of the data to be considered, including animal‐, testis‐, and spot‐level sources of variation.

However, the study is not without its limitations. First, simultaneous semen evaluation, endocrine profiling, histopathological examination, and Doppler ultrasonography were not performed; therefore, the functional significance and relation to fertility of the observed NPV differences could not be directly determined. Second, the study was conducted in a single breed under specific environmental and management conditions, which may limit direct extrapolation to other ram breeds or production systems. Third, the focal depth was maintained at a constant machine setting rather than being repositioned relative to the mediastinum in each testis. Although this prevented operator‐dependent adjustment of the focal setting among animals and sampling periods, it may have contributed to depth‐dependent differences in NPV. Moreover, although ROI placement and imaging plane were systematically evaluated, the study was not designed to determine the precise histological or acoustic mechanisms underlying the marked differences between above‐ and below‐mediastinum measurements. The difference between the regions above and below the mediastinum may reflect not only regional parenchymal heterogeneity but also differences in depth (sampling location), beam path, attenuation, time‐gain compensation, and focal‐zone proximity. In addition, the greater mean NPV observed in older rams should not be interpreted as proof of improved testicular function or as evidence of a specific histological process. Although no clinically or ultrasonographically overt lesions were identified in the included animals, histopathological examination was not available, and microscopic fibrosis, mineralization, or other subclinical age‐related changes cannot be excluded. Future studies integrating echotexture, Doppler ultrasonography, semen quality, endocrine profiling, and, where feasible, histological validation in the same rams across different reproductive periods and age categories would be especially valuable for determining whether the substantial NPV differences observed here can be translated into a more comprehensive assessment of breeding soundness. Another limitation of the study may be the only two examinations performed in August and March. The study did not include repeated measurements during seasonal regression, transition, or recrudescence. Therefore, the results describe a within‐animal difference between two predefined reproductive‐period sampling points rather than the complete annual trajectory of testicular echogenicity. Monthly or more frequent longitudinal examinations, ideally combined with endocrine, semen, and environmental measurements, are required to characterize the nonlinear seasonal dynamics of testicular NPV.

In conclusion, under the fixed acquisition conditions of the present study, testicular parenchymal NPV was strongly influenced by sampling location relative to the mediastinum and, to a lesser extent, by imaging plane. NPV also differed among age groups and between the August and March examination periods. Because only two sampling points were evaluated, these findings should not be interpreted as describing the complete seasonal trajectory of testicular echogenicity. Rather, they demonstrate that ROI location, imaging plane, focal position, and sampling period must be explicitly standardized and reported before NPV can be compared across animals or studies. That is, although this study does not validate NPV as a fertility biomarker, it demonstrates that NPV is highly sensitive to ROI placement and imaging plane, and this methodological insight should be taken into account in future ultrasonographic investigations of the ram testis. In addition, future standardized protocols should position the focal zone relative to an anatomical landmark, preferably at or just below the mediastinum testis, and should report the focal depth together with gain and ROI depth.

## Author Contributions

Bülent Bülbül, Şükrü Doğan and Mustafa Yiğit Nizam conceptualized and designed the study. Bülent Bülbül and Necdet Akay conducted fieldwork and data collection. Şükrü Doğan performed statistical analysis. Bülent Bülbül and Mustafa Yiğit Nizam wrote the draft and edited the manuscript.

## Funding

Funding for this research was provided by the Republic of Turkey Ministry of Agriculture and Forestry, General Directorate of Agricultural Research and Policies (TAGEM). Open access payment was covered by: TUBITAK ULAKBIM.

## Disclosure

During the preparation of this manuscript, the authors used OpenAI ChatGPT (version 5.0) solely for language editing. All content was subsequently reviewed and revised by the authors, who assume full responsibility for the accuracy and integrity of the final publication.

## Ethics Statement

This study used data and ultrasonographic images obtained during routine pre‐breeding health examinations of rams. Based on Article 8, Section 8‐k2 of the ‘Regulation on the Working Procedures and Principles of Animal Experiments Ethics Committees’ (Official Gazette No. 28914, February 2014), which specifies that clinical applications performed for diagnostic and therapeutic purposes—such as routine health screenings—fall outside the scope of HADYEK (Local Ethics Committee for Animal Experiments) approval, the Ethics Committee for the Use of Animals in Research and Experimentation at the Bahri Dağdaş International Agricultural Research Institute (Turkey) determined that formal ethical approval was not required for this study. The authors also complied with the ARRIVE guidelines.

## Conflicts of Interest

The authors declare no conflicts of interest.

## Supporting information


**Table S1:** The EMMEANS of NPVs for AgeGroup × Season × ROI interactions.

## Data Availability

The data of this study are available from the corresponding author (B. Bülbül) upon reasonable request.
