# Supplementary material for: Methodological Determinants of Grayscale Pixel Intensity in Ram Testicular Ultrasonography: Effects of ROI Placement and Imaging Plane Across Age Groups and Two Reproductive Periods
Source: Reprod Domest Anim. 2026 Aug 4;61(8):e70302. doi: 10.1111/rda.70302 (PMC13435824; doi:10.1111/rda.70302)
Supplement: Supplementary file 1 [file RDA-61-e70302-s001.docx]

**Methodological determinants of grayscale pixel intensity in ram testicular ultrasonography: effects of ROI placement and imaging plane across age groups and two reproductive periods**

**Table S1.** The EMMEANS of NPVs for AgeGroup × Season × ROI interactions.

| **Factor / Interaction** | **Mean NPV** | **SE** | **95% CI** |
| --- | --- | --- | --- |
| **AgeGroup × Season × ROI** |  |  |  |
| Group 1 × Breeding × Above | 39.08^h^ | 3.42 | 32.13 - 46.04 |
| Group 1 × Breeding × Below | 82.85^d^ | 3.42 | 75.89 - 89.80 |
| Group 1 × Non-breeding × Above | 41.14^h^ | 3.42 | 34.18 - 48.09 |
| Group 1 × Non-breeding × Below | 79.14^e^ | 3.42 | 72.18 - 86.10 |
| Group 2 × Breeding × Above | 43.63^g^ | 2.79 | 37.95 - 49.31 |
| Group 2 × Breeding × Below | 97.28^b^ | 2.79 | 91.60 - 102.96 |
| Group 2 × Non-breeding × Above | 35.41^g^ | 2.79 | 29.73 - 41.09 |
| Group 2 × Non-breeding × Below | 77.36^e^ | 2.79 | 71.68 - 83.04 |
| Group 3 × Breeding × Above | 54.11^f^ | 3.23 | 47.55 - 60.66 |
| Group 3 × Breeding × Below | 112.94^a^ | 3.23 | 106.38 - 119.49 |
| Group 3 × Non-breeding × Above | 44.85^g^ | 3.23 | 38.29 - 51.41 |
| Group 3 × Non-breeding × Below | 87.46^c^ | 3.23 | 80.90 - 94.01 |

^a,b,c,d,e,f,g,h^Within each panel, means with different superscript letters differ significantly (Bonferroni-adjusted p < 0.05).

EMMEANS: estimated marginal means, NPV: numerical pixel value, ROI: region of interest
